# Aconite in Headache

**Published:** 1874-10

**Authors:** 


Aconite in Headache.—Dr. Fothergill (*British Medical Journal* January 10, 1874,) recommends the use of aconite in congestive headaches, attended with great cotemporary coldness of the hands and feet. The remedy dilates the peripheral blood-vessels, and so relieves the congested cerebral vessels.

